# Reactive Arthritis Unmasked by Tuberculosis: A Rare Case of Poncet's Disease With Disseminated TB in a Malnourished Young Adult

**DOI:** 10.1002/ccr3.71450

**Published:** 2025-11-09

**Authors:** Kanza Farhan, Nayab Magsi, Maheen Afaq, Faraz Shafi, Ahmed Asad Raza, Abedin Samadi

**Affiliations:** ^1^ Sindh Medical College Jinnah Sindh Medical University Karachi Pakistan; ^2^ Sindh Employees Social Security Institution Karachi Pakistan; ^3^ Kabul University of Medical Science Abu Ali Sina Kabul Afghanistan

**Keywords:** extra pulmonary, malnutrition, Poncet's disease, reactive arthritis, tuberculosis

## Abstract

Poncet's disease is a rare, non‐destructive reactive arthritis that occurs in the presence of active tuberculosis (TB) without direct joint infection. It is often underrecognized in TB‐endemic regions due to its clinical overlap with septic or autoimmune arthritis. We report the case of a 22‐year‐old undernourished female from rural Pakistan who presented with a 4‐week history of bilateral knee pain and swelling, low‐grade fever, dry cough, appetite loss, and weight loss. Physical examination revealed joint swelling without erythema, bilateral lung crepitations, and a BMI of 15.7 kg/m^2^. Initial laboratory investigations showed anemia, elevated ESR and CRP, while autoimmune and infectious screens were negative. Synovial fluid analysis from the knee was sterile and non‐purulent. Chest X‐ray and GeneXpert confirmed active pulmonary TB. Abdominal imaging showed hepatic parenchymal changes and moderate ascites. Based on clinical, radiological, and serological findings, a diagnosis of Poncet's disease was made in accordance with Sharma's diagnostic criteria. The patient was initiated on standard anti‐tubercular therapy (HRZE) and supportive nutritional management. Non‐steroidal anti‐inflammatory drugs (NSAIDs) were used initially. Marked clinical improvement was observed within 3 weeks, including resolution of joint pain and systemic symptoms. At follow‐up, she showed weight gain and normalized joint function without relapse. This case highlights the importance of recognizing Poncet's disease as a differential diagnosis in patients with unexplained arthritis in TB‐prevalent areas. Early identification can prevent misdiagnosis and avoid unnecessary use of immunosuppressants or prolonged antibiotics. Poncet's disease should be considered in patients with inflammatory arthritis and active TB, especially when synovial cultures are negative. Awareness and adherence to diagnostic criteria can aid in timely diagnosis and management.

**Summary**

- Poncet's disease, a rare reactive arthritis associated with active tuberculosis, should be suspected in TB‐endemic areas when patients present with sterile inflammatory arthritis.
- Early recognition, guided by diagnostic criteria, allows prompt anti‐tubercular therapy, avoids unnecessary immunosuppression, and leads to complete recovery without joint damage.

## 1 Introduction

Tuberculosis (TB) remains a major global health concern, particularly in low and middle‐income countries such as India, Pakistan, Nigeria, Bangladesh, and Ethiopia. It is one of the leading causes of death from infectious diseases, responsible for more than 1.2 million deaths annually and affecting over 10 million people worldwide [1]. Pulmonary TB is the most common form. Extrapulmonary disease, especially disseminated TB involving the liver, spleen, kidneys, or central nervous system, may present with atypical and severe manifestations, particularly in immunocompromised individuals [2].

Musculoskeletal TB is relatively uncommon and accounts for only 1% to 3% of all TB cases [1]. Among its manifestations, Poncet's disease is an unusual form of non‐septic, reactive polyarthritis associated with active TB in which mycobacteria are absent from the affected joints [3]. Because its presentation often overlaps with other rheumatologic disorders, failure to consider it in the differential diagnosis may delay recognition and treatment, especially in TB‐endemic regions [4].

We report a rare case of Poncet's disease in a 22‐year‐old underweight female with acute pulmonary TB and disseminated features including abdominopelvic ascites and altered hepatic echotexture. This case highlights the importance of early recognition of TB‐related reactive arthritis in order to ensure timely diagnosis and appropriate therapy.

## 2 Case Presentation

### 2.1 Patient Information

A 22‐year‐old unmarried lady from a rural region of Pakistan presented to the outpatient department with joint swelling and pain persisting for 4 weeks, accompanied by additional symptoms. She stated that she had no identified comorbidities or a history of tuberculosis. She was in a household with several inhabitants and few financial resources. No history of drug, alcohol, or tobacco use was present. Her familial medical history was noncontributory, and she was not on any medications upon admission.

### 2.2 Chief Complaints

The primary issues included bilateral knee pain and swelling persisting for 4 weeks, intermittent low‐grade fever, generalized weakness, diminished appetite, a dry cough, and a weight loss of 5 kg over the prior 2 months.

### 2.3 Clinical Findings

The patient consistently appeared ill, exhibited underweight status (BMI 15.7 kg/m^2^), and displayed pallor. During the examination, her vital signs were stable and she exhibited no temperature. The musculoskeletal evaluation revealed edema, pain, and restricted mobility in both knees, along with some swelling in the left ankle joint. The joints exhibited neither warmth nor erythema. The respiratory examination revealed bilateral crepitations at the lung bases. No cutaneous rashes, lymphadenopathy, or neurological issues were present.

### 2.4 Timeline

A comprehensive timeline summarizing the progression of clinical symptoms, key diagnostic evaluations, initiation of treatment, and subsequent therapeutic response is provided below (Table 1). This chronological overview facilitates a clearer understanding of the patient's clinical course and supports the diagnosis of Poncet's disease in the context of disseminated tuberculosis.

**TABLE 1:** Timeline outlining the patient's symptom progression, diagnostic steps, initiation of anti‐tubercular therapy, and clinical recovery consistent with Poncet's disease.

Timeframe	Clinical event
6 weeks before visit	Onset of joint pain and swelling
5 weeks before visit	Low‐grade fever, cough, weight loss
Day 1 (Presentation)	Hospital visit, clinical exam, lab and imaging workup
Day 3	Confirmed diagnosis of pulmonary TB and reactive arthritis (Poncet's disease)
Day 4	Initiated anti‐tubercular therapy (HRZE)
Week 3	Resolution of joint symptoms and fever
Week 7	Weight gain, no joint limitations, improved imaging

### 2.5 Diagnostic Assessment

The initial laboratory tests indicated microcytic hypochromic anemia (Hb 8.9 g/dL), an elevated erythrocyte sedimentation rate (ESR: 76 mm/h), and an increased C‐reactive protein level (CRP: 32 mg/L). The counts of leukocytes and assessments of hepatic and renal function were all within normal ranges.

Rheumatoid factor (RF), anti‐cyclic citrullinated peptide (anti‐CCP), antinuclear antibodies (ANA), anti‐smooth muscle antibodies (ASMA), and anti‐mitochondrial antibodies (AMA) were all negative for autoimmune markers.

**FIGURE 1:**
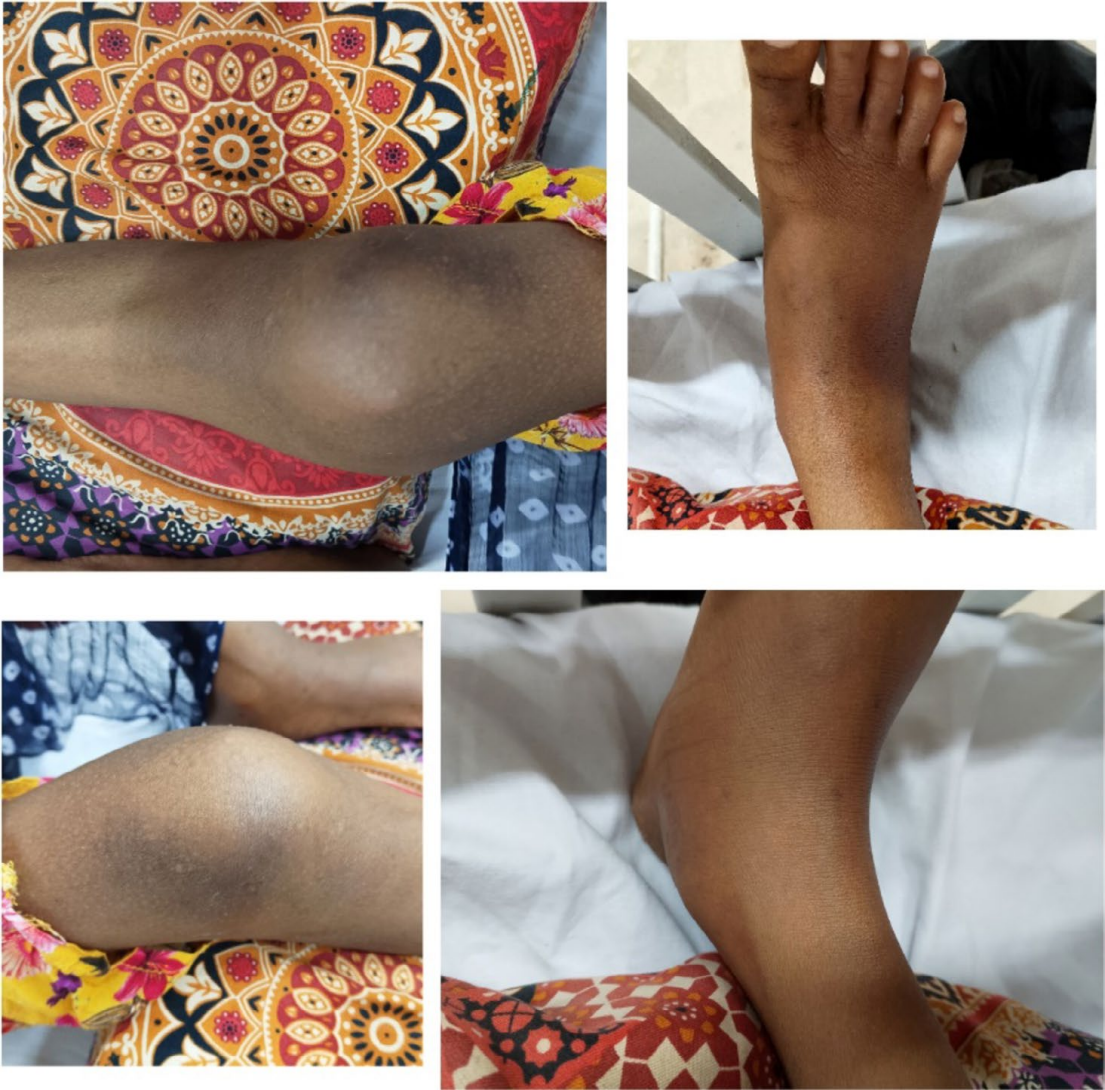
Swollen, warm, and tender right knee and ankle joints without erythema.

The testing for HIV, hepatitis B, and hepatitis C viruses was also negative. The Mantoux test yielded a positive result, exhibiting an induration of 18 mm. A chest X‐ray revealed patchy bilateral infiltrates suggestive of active pulmonary tuberculosis (Figure 1). The sputum smear for acid‐fast bacilli (AFB) tested positive, and the GeneXpert assay confirmed the presence of *Mycobacterium tuberculosis* , which exhibited no resistance to rifampicin.

**FIGURE 2:**
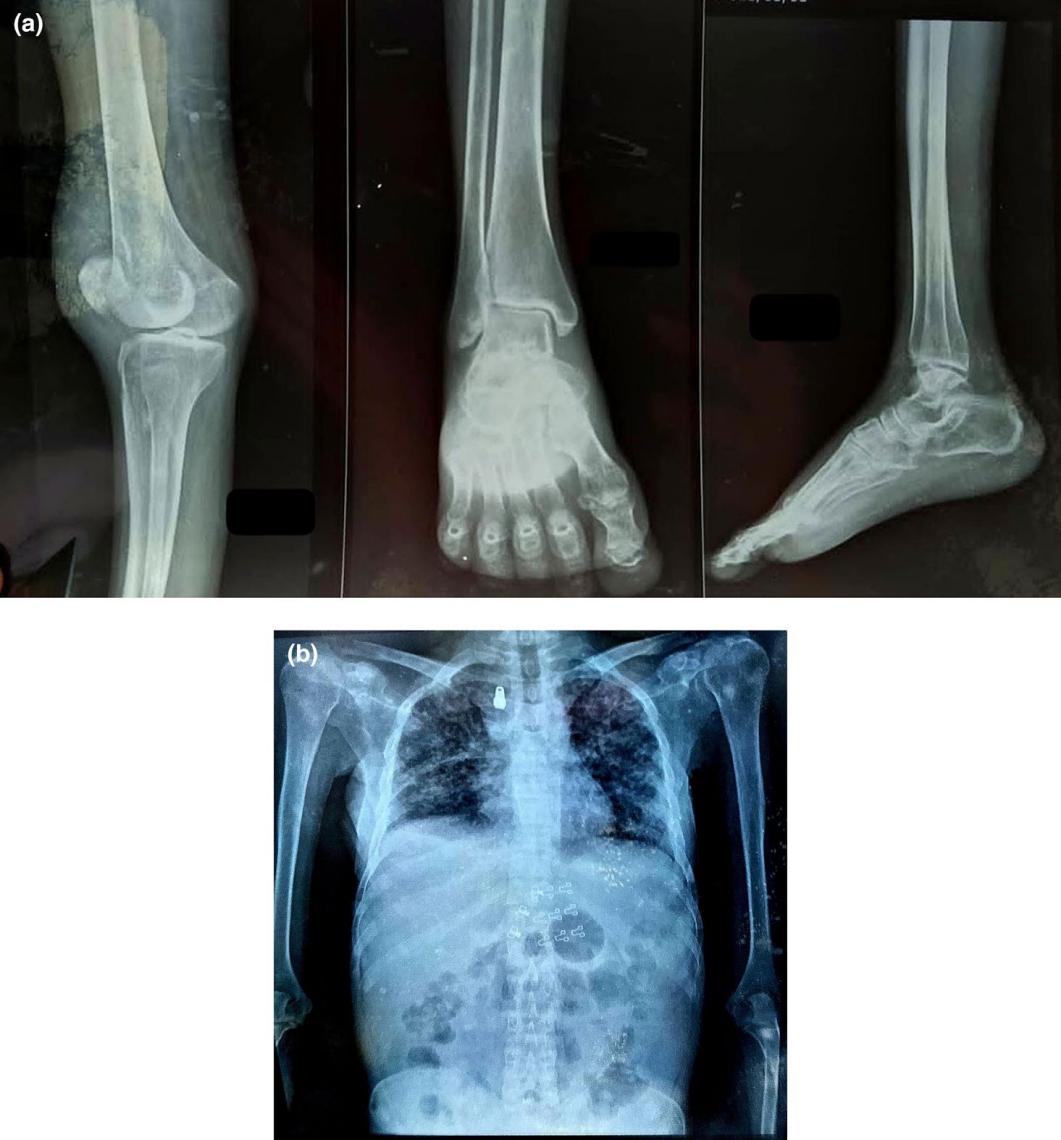
(a) Joint X‐ray showing no significant abnormalities in the knee and ankle joints. (b) Chest X‐ray showing increased opacity in both upper zones, predominantly on the right side.

**FIGURE 3:**
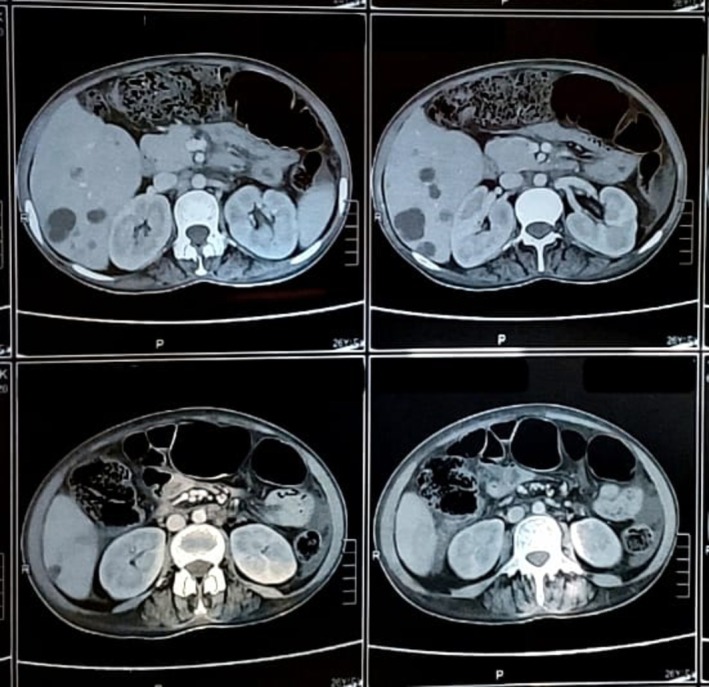
Abdominal CT scan showing nonhomogeneous liver texture and mild abdominopelvic ascites.

An abdominal ultrasound revealed a coarse, irregular hepatic echotexture and significant ascites in the belly and pelvis (Figure 2a,b). The knee MRI revealed a minor accumulation of fluid in the joints and thickening of the synovial membrane, with no evidence of erosions or bone injury (Figure 3).

The arthrocentesis yielded sterile, straw‐colored synovial fluid including a significant quantity of lymphocytes. No creatures or crystals existed, and the cultures remained unfavorable. Blood cultures and echocardiography excluded endocarditis.

The diagnosis of Poncet's disease, a reactive arthritis resulting from active tuberculosis, was established based on the patient's symptoms, sterile inflammatory arthritis, evidence of active pulmonary tuberculosis, and the exclusion of other infectious or autoimmune etiologies, in accordance with the diagnostic criteria proposed by Sharma et al. [5] (Table 2).

**TABLE 2:** Diagnostic criteria for Poncet's disease proposed by Sharma et al. [5].

Category	Criteria
Essential criteria	Evidence of inflammatory arthritis that is non‐erosive and non‐deforming. Other causes of inflammatory arthritis have been reasonably excluded.
Major criteria	Confirmed diagnosis of extra‐articular tuberculosis. Complete clinical resolution following anti‐tubercular therapy.
Minor criteria	Positive Mantoux test (Tuberculin Skin Test) Presence of TB‐related hypersensitivity signs (e.g., erythema nodosum, phlyctenular keratoconjunctivitis). No signs of sacroiliac or axial joint involvement.
Classification	Definitive: Essential +2 Major Probable: Essential +1 Major +3 Minor Possible: Essential +1 Major +2 Minor/Essential +3 Minor

### 2.6 Therapeutic Intervention

The patient commenced standard first‐line anti‐tubercular therapy (HRZE: isoniazid, rifampicin, pyrazinamide, and ethambutol) at dosages determined by their weight. Nutritional therapy and multivitamin supplementation constituted components of supportive treatment. No corticosteroids or any immunosuppressive agents were administered. NSAIDs were administered during the initial phase when symptoms manifested.

### 2.7 Follow‐Up and Outcomes

By the third week of treatment, the patient's clinical state had markedly improved, with the resolution of fever and joint issues. The individual's appetite and weight continued to improve. After 2 months, she had regained 3.5 kg, her joints were devoid of pain and exhibited full range of motion, and a subsequent chest X‐ray verified the resolution of the infiltrates. A subsequent ultrasound revealed a significant reduction in ascites. She adhered to her treatments and completed the intensive phase of tuberculosis treatment without any complications.

## 3 Discussion

Our patient initially presented with oligoarthritis and was later diagnosed with active disseminated tuberculosis (TB). Oligoarthritis refers to inflammation involving two to four joints and, when acute, is considered a rheumatologic emergency. The most important causes include septic arthritis, acute gout, pseudogout, reactive arthritis, and the initial phase of polyarthritis [6, 7].

In developing countries such as Pakistan, India, and Bangladesh, TB is an important differential diagnosis for patients with multisystem involvement. According to the World Health Organization (WHO), Pakistan, along with India, Indonesia, China, and the Philippines, accounted for more than half of global TB cases in 2023, contributing to a record high of 8.2 million newly diagnosed cases worldwide [8].

Joint involvement in TB can manifest as tuberculous arthritis or Poncet's disease. Tuberculous arthritis usually affects a single joint and results from direct invasion by *Mycobacterium tuberculosis* , most often via hematogenous spread from a primary site, though contiguous spread or direct inoculation may also occur. The bacillus can often be cultured from the affected joint. In contrast, Poncet's disease is a non‐infectious, inflammatory arthritis involving one or a few joints. It typically arises during the acute phase of TB and represents a reactive, aseptic process [3, 5, 9].

This case is notable for its oligoarticular involvement accompanied by hepatic dysfunction, ascites, hematological abnormalities, and nutritional deficiency, pointing toward disseminated TB. The absence of organisms in synovial fluid, negative autoimmune markers, and the patient's rapid improvement with antitubercular therapy strongly supported the diagnosis of Poncet's disease [5]. As a rare form of reactive arthritis, it can mimic septic or autoimmune arthritis, making timely recognition particularly challenging in TB‐endemic settings. Our patient, a 22‐year‐old female, initially received broad‐spectrum antibiotics and anti‐inflammatory therapy, but only limited benefit was observed. Marked clinical improvement occurred after disseminated TB was confirmed by positive sputum culture and appropriate antitubercular therapy was initiated.

Few reports have described the simultaneous occurrence of tuberculous septic arthritis and Poncet's disease affecting different joints [10, 11]. Therefore, it remains essential to exclude bacillary invasion of the affected joint by performing synovial fluid microscopy and culture. The pathogenesis of Poncet's disease is still incompletely understood, but molecular mimicry involving mycobacterial heat shock proteins, particularly Mtb‐Hsp60 and Mtb‐Hsp65, has been proposed as a key mechanism [12]. This case also emphasizes the need for closer collaboration between rheumatologists, infectious disease specialists, and internists, as timely recognition of Poncet's disease requires a multidisciplinary perspective. Early recognition not only prevents unnecessary interventions but also facilitates prompt initiation of antitubercular therapy, which remains the cornerstone for both systemic and joint recovery.

## 4 Conclusion

To conclude, active tuberculosis should always be considered as a possible underlying cause in patients presenting with unexplained arthritis and fever, particularly in regions where the disease is highly prevalent. The diagnosis is often difficult and requires a high degree of clinical suspicion together with appropriate investigations. Because many healthcare providers are unfamiliar with Poncet's disease, this condition is frequently overlooked or misinterpreted as another rheumatologic or infectious disorder. As a result, patients may be subjected to unnecessary therapies, including broad‐spectrum antibiotics, which not only delay effective treatment but may also contribute to the growing problem of antimicrobial resistance. Greater awareness and timely recognition are therefore essential to avoid misdiagnosis, reduce inappropriate interventions, and ensure favorable outcomes through prompt initiation of antitubercular therapy.

## 5 Patient's Perspective

“I had constant joint pain and couldn't walk right before I came to the hospital. I also felt weak and lost a significant amount of weight. I thought I just had a fever, but when they told me it was tuberculosis and something wrong with my joints, I was startled. After beginning treatment, I felt a lot better. I can now move around and eat normally again. I'm grateful to the doctors for detecting the illness early.”

## Author Contributions

**Kanza Farhan:** conceptualization, project administration, supervision, visualization, writing – original draft, writing – review and editing. **Nayab Magsi:** formal analysis, investigation, validation, writing – original draft, writing – review and editing. **Maheen Afaq:** methodology, writing – original draft, writing – review and editing. **Faraz Shafi:** project administration, writing – original draft, writing – review and editing. **Ahmed Asad Raza:** writing – original draft, writing – review and editing. **Abedin Samadi:** project administration, writing – review and editing.

## Ethics Statement

Ethical approval was not required for this single case report in accordance with the policy of our institution.

## Consent

The patient provided written consent for the publication of this case report and any accompanying images.

## Conflicts of Interest

The authors declare no conflicts of interest.

## Data Availability

The datasets generated and/or analyzed during the current study are not publicly available due to patient confidentiality but are available from the corresponding author on reasonable request.
